# Do payments for forest ecosystem services generate double dividends? An integrated impact assessment of Vietnam’s PES program

**DOI:** 10.1371/journal.pone.0200881

**Published:** 2018-08-01

**Authors:** Thu-Ha Dang Phan, Roy Brouwer, Long Phi Hoang, Marc David Davidson

**Affiliations:** 1 Institute for Biodiversity and Ecosystem Dynamics, University of Amsterdam, Amsterdam, the Netherlands; 2 Department of Environmental Economics, Institute for Environmental Studies, VU University Amsterdam, Amsterdam, the Netherlands; 3 Department of Economics and the Water Institute, University of Waterloo, Waterloo, Canada; 4 Water Systems and Global Change Group, Wageningen University, Wageningen, the Netherlands; Chinese Academy of Forestry, CHINA

## Abstract

Payments for ecosystem services (PES) often serve multiple objectives, such as carbon emission reduction and poverty alleviation. However, the effectiveness of PES as an instrument to achieve these multiple objectives, in particular in a conservation-development context, is often questioned. This study adds to the very limited empirical evidence base and investigates to what extent Vietnam’s move to PES has helped protect forest ecosystems and improve local livelihoods and income inequality. We zoom in on Lam Dong province, where PES was first introduced in Vietnam in 2009. Changes in forest cover are analysed using satellite images over a period of 15 years (2000–2014). Socio-economic impacts are assessed based on rural household interviews with PES participants and non-participants as a control group over a period of 7 years (2008–2014). Our results show that PES contributes significantly to forest cover, the improvement of local livelihoods, and the reduction of income inequality.

## Introduction

Ecosystems provide multiple benefits to humanity, ranging from provisioning services such as food and water to supporting and regulating services such as nutrient cycling and carbon sequestration [1]. Since many of these services are public goods, the private owners of the land on which ecosystems are situated often lack incentives to protect them. As a result, ecosystem services protection is less than optimal from a societal point of view. A variety of policy options are available to protect the world’s ecosystems and the services they provide, of which designated protected areas are probably the most important [2]. In the last decade, however, there has been increasing interest in market-based instruments such as Payments for Ecosystem Services (PES) [3–5]. In this study we employ the revised definition of PES [6] as “voluntary transactions between service users and service providers that are conditional on agreed rules of natural resource management for generating offsite services” to better reflect the wide variety of payment schemes in practice [7]. PES is considered an important policy instrument to achieve environmental objectives while at the same time addressing equity concerns and alleviating poverty, especially in developing countries. Indications of PES success are often seen as a combination of the provision of a critical ES together with livelihood, social capital or institutional capacity improvements [8, 9] and many PES programs therefore actively involve poor farmers [10–17]. However, the effectiveness of achieving both objectives with one and the same policy instrument has been questioned. There are usually significant trade-offs between nature conservation and economic development. The involvement of poor farmers, often without legal land entitlements and hence incentives to invest in the quality of the land they use, may contribute only marginally to improving ecosystems and the services they provide.

In practice it has proven very difficult to quantify the environmental and socio-economic impacts of PES, as PES is often embedded in a policy mix and part of broader socio-economic development trends [3, 11, 15]. While not taking into account additionality and leakage as two of the most challenging environmental variables to measure can undermine both the economic and environmental efficiency of PES schemes [18, 19], there are also challenges in measuring changes in ecosystem services provision, owing to a systematic lack of information and international monitoring guidelines [5]. Not surprisingly, studies on PES effectiveness consequently report diverging results which seem to be highly context-dependent [20]. For instance, PES impacts on deforestation in tropical countries are highly case-dependent, ranging from no improvement at all (e.g. [21–24] or substantial improvements (e.g. [25]). Moreover, prioritizing the provision of ecosystem services does not automatically guarantee optimal biodiversity conservation and vice versa [26]. The behavioural effect of PES on participating land owners and their management practices is not always clear [27], while documentation of its impact on participants’ livelihoods is also largely lacking. Studies examining the economic effects of PES on household prosperity show a similar trend, with both positive and negative changes in reported income levels (e.g. [28–31]). For example, [32] examined 4 case studies in Central America to assess other PES co-benefits and found that PES payments can positively contribute to household and community well-being through the provision of material household needs and capacity building, such as forest management training. To date, there has been little research that simultaneously quantifies the socio-economic and environmental consequences of PES (e.g. [33–34]).

Given the contested views and sometimes contradictory empirical evidence of the effectiveness of PES in delivering both ecosystem protection and improved livelihoods, the purpose of this study is to add to the limited empirical evidence base and quantify the impacts of PES on forest ecosystems, household livelihoods and income levels in Lam Dong province, Vietnam, where PES was first introduced in 2009. As noted by [35] there is an absence of clear evidence that PES in Vietnam is indeed helping to maintain and improve the environment, while social impacts of PES appear to be mixed due to a lack of available credible data. In general, [36] observe that studies tend to fail to report even basic information related to, for example, the actual amount PES participants are paid and how often they get paid. This information is essential in order to be able to assess the incentive-compatibility of the payment structure, as well as the socio-economic impacts of the payments on the sustainability of the livelihoods, and will therefore be an explicit component of the data and information collected in this study. To this end we carry out an integrated environmental-economic impact assessment and analyse changes in forest cover using MODIS satellite images over a period of 15 years (2000–2014) and conduct in-depth rural household interviews with PES participants and non-participants to assess the impact of PES over a period of 7 years (2008–2014), before and after the introduction of PES in the study area.

## Background

Since the 1990s the Vietnamese government has implemented several national forest programs, including payments for participating farmers, such as Program 327 in 1992 and Program 661 in 1998. Continuation of these state funded programs proved difficult, however, owing to a lack of long-term funding and low payment rates, ranging from US$2.5 to 5.0 per ha per year for Programs 327 and 661, respectively [37]. PES was therefore considered an attractive policy alternative in view of the fact that funding is generated through a market-liked mechanism, from both state owned and private stakeholders such as water supply companies, hydropower plants, and tour operators in the tourism sector.

In 2009, two PES pilot projects were implemented in the provinces Lam Dong and Son La as preambles to a national PES program that was officially launched in 2011. Current PES policy in Vietnam focuses primarily on ecosystem services such as water supply regulation, soil conservation and landscape beauty. It is also important to note that the PES schemes in Vietnam are not as voluntary as defined by [6]. In fact, there exists “a regulatory driver” on both the demand and supply side [9] i.e. the Vietnamese government is involved in setting payment levels and selecting ES buyers and sellers [35]. Also, the country’s PES policy tends to prioritize the involvement of poor and often ethnic-minority farmers [35, 37]. However, this is not uncommon in this field [3, 16], and more importantly, the PES scheme meets the important conditionality criterion. While ES sellers are primarily selected by the government, participating farmers as ES providers decide voluntarily whether or not they want to participate in the PES scheme. The focus in this study is furthermore not on whether Vietnam’s PES scheme fully reflects the original definition of PES [14], but rather whether the payment scheme has contributed to local poverty alleviation and forest conservation.

This study focuses on the implementation of PES in Lam Dong province as one of the oldest PES schemes in Vietnam. The scheme was implemented under joint supervision of several governmental agencies, with the Lam Dong Forest Protection and Development Fund (FPDF) as program intermediary. ES buyers and beneficiaries are 10 water supply companies, 14 hydropower plans and 12 tour operators [38]. ES sellers as official forest owners include 9 state-owned forest companies, 38 private companies, 16 forest management boards, 4 national parks and close to 1,800 individual households. In most cases, forest owners sign forest protection contracts with local farmers, preferably with those who have experience with forest protection and were involved in forestry programs in the past, making the latter the actual ES providers. Currently more than 12,000 such contracts with local households have been concluded. The FPDF collects payments from the ES buyers and distributes this among the forest owners (i.e. ES sellers) based on their forest area, who in turn pay the local farmers once every three months.

In Lam Dong province, the Don Duong district was chosen as our study area in direct consultation with the FPDF and the local authority. The district is located in the Da Nhim watershed [38]. Compared with surrounding districts Don Duong has a representative share of PES participants (11% of total district’s population) [39, 40]. The district is also representative in terms of ethnic-minority groups (30% of the households belong to an ethnic minority) [39]. Within the district of Don Duong, four villages were selected, which are similar in terms of their bio-physical and socio-economic characteristics (for more details, see S1 Table) and have a representative mix of ethnic backgrounds. The total forested area under PES in these villages is 9,728 ha. The forests in the study area are classified as production and protected forest, with the latter being the dominant forest type [39]. The main livelihoods are agriculture (coffee, fruit and vegetables), forestry, hired labour, and trade.

## Methodology

### Household survey

Both scheme participants and non-participants were selected randomly from the four villages in the absence of any prior demographic or socioeconomic census information. Overall, we aimed to include in our survey (i) respondents who are older than 18 years, (ii) a balanced mix of both the majority (Kinh) and minority ethnical groups, and (iii) respondents who are currently involved in agriculture as farmers, and if possible with past experience in forestry activities. In the end, two hundred and sixty PES participants were sampled randomly based on a list of participating households provided by the local Forest Management Board. One hundred and fifteen non-participants were randomly selected in four villages based on consultations with the local authorities. Interviewing took place over three weeks in October 2014 with the help of a structured, thoroughly pretested questionnaire. The response rate was 81%, yielding 303 interviews in total of which 209 households participate in the PES scheme.

The questionnaire consists of five main parts (see S1 Text and S2 Text). The first part asks respondents warm-up questions about their socio-demographic household characteristics such as the age of the household head, education level, number of family members, etc. The second part asks respondents about their specific past and current agriculture and forestry activities. The third part is devoted to the household’s income sources and household expenditures, both before and after participation in the PES scheme. Here both quantitative (e.g. monetary income flows) and qualitative (e.g. respondent’s reporting of income sources and wealth) data and information was collected and used to assess the socioeconomic status of the interviewed households. Respondents were asked to list all income sources, including income generated from daily employment, government subsidies, pensions, and so on. All these income sources were also quantified and measured in monetary units. Scheme participants were asked about their income sources and income amounts before and after their participation in the PES scheme, while non-participants were asked about their past and current income sources with no reference to the PES scheme. More qualitative information was elicited by asking all respondents how they perceived socio-economic conditions and possible changes over time in their household’s welfare and well-being. Respondents were furthermore questioned about any possible changes in their level of environmental awareness over time. Where possible, the answers to these more qualitative questions were converted into a numerical format. The fourth part zooms in on the costs incurred by farmers in both agriculture and forestry activities and the revenues from these activities. In the fifth and final part participating respondents are asked to indicate in a more qualitative manner how the PES scheme has impacted family welfare, environmental awareness and forest protection. Interviews lasted between 45 and 60 minutes.

The household questionnaire was developed in close consultation with local forestry researchers, and thoroughly reviewed and pre-tested by four experienced staff members from the Forest Protection and Development Fund (FPDF) in Lam Dong. Most of the questions were straightforward, asking respondents about the demographic and socio-economic characteristics of their household and their income generating activities. Both the review and pre-test yielded useful feedback and recommendations for changes in some of the questions included in the questionnaire to better accommodate specific local conditions and circumstances in the case study area, such as crop types. In addition, during the main survey, the hired local interviewers were debriefed at the end of every interview day and the completed questionnaires were checked by the lead author of this paper in case relevant data and information was missing. The answers to the questions in the questionnaire were converted as much as possible into a numerical format and entered into an Excel database, which was in turn checked and screened as well to ensure data were coded correctly and consistently. Qualitative data were translated into English and also entered in the same database. Finally, the Excel database was exported into Stata for statistical data analysis. More details about the database can be found in S1 File.

In addition to the household survey, two in-depth interviews were conducted with the head of the local forest management board in Dran and the director of the FPDF in Lam Dong. The results coming out of these interviews served as supplementary information, especially to qualitatively underpin the results of the environmental impact assessment.

### Econometric models

A Full Information Maximum Likelihood (FIML) treatment effects regression model was employed to examine the determinants of income change for households who participated and households who did not participate in the PES scheme in order to address possible endogeneity [41]. PES participation is a treatment variable, implying that it is included in a set of simultaneously estimated equations both as a dependent and independent variable to explain household income change, whilst vice versa the factors that determine the decision to participate includes household income.

The treatment effects model predicts the likelihood of a farmer’s participation in PES using a probit regression model and a linear Ordinary Least Square (OLS) regression model to analyse the (*relative*) household income change. In the probit selection model (Eq 1), the dependent variable is defined as whether or not a household participates in PES (*PES*_i_ = 1 if the household participates and *PES*_i_ = 0 otherwise). Variables entering the regression model are grouped into: *General household characteristics* (ethnicity, age, years living in the study area, distance to forest, dependent family members), *Income before PES*, and *Pre-PES forestry activities*. Ethnicity was included as a regressor given the prioritization to poorer, ethnic minority farmers. Due to a significant positive correlation between ethnicity and education level, the latter variable was not included in the regression model. Both distance to forest and pre-PES income have been shown to negatively impact PES participation in a previous study in Mexico [29]. The number of financially dependent family members is expected to correlate positively with household willingness to participate, as well as previous involvement in forestry activities, which is expected to positively influence households’ experience and familiarity with PES activities.

$${PES}_{i}=\beta_{01}+{\beta^{GEN}General}_{i}+\beta^{INC}{PrePESincome}_{i}+{\beta^{FOR}Forestry}_{i}+\varepsilon_{i}$$ (1)

$${Income}_{i}=\beta_{02}+{\beta^{GEN}General}_{i}+\beta^{LAB}{Labour}_{i}+\beta^{AGR}{Agriculture}_{i}{+\beta^{PES}PES}_{i}+\omega_{i}$$ (2)

In the OLS regression (Eq 2), the dependent variable *Income_i_* is defined as the *relative* change in household income before and after PES implementation. The explanatory variables are again grouped as before into *General household characteristics*, *Agriculture* (land area and main crop), *Labour* (number of working, and dependent members), and *PES* (whether or not the household participates in PES; and for how long they have been participating. We predicted a positive relationship between income change and belonging to the majority ethnic group and years living in the study area. We added land area and crop type since these factors drive agricultural income in particular and total income in general. The theoretical expectation regarding the number of working family members is that it correlates positively with income change. Lastly, the dependent variable PES participation in Eq 1 and the number of years in PES are expected to correlate positively as two of the explanatory variables in Eq 2 with changes in household income as a result of the payments involved. *β* is the vector of effect estimators, with the superscript referring to the group of regressors to which the estimated coefficient belongs. *β*_01_ and *β*_02_ are the intercepts and *ω_i_* and *ε_i_* are the vectors of residual terms. Both *ω_i_* and *ε_i_* are assumed to be normally distributed with mean zero and variance $\sigma_{\omega }^{2}$ and $\sigma_{\varepsilon }^{2}$, respectively. The treatment effects model assumes that the level of correlation between these two error terms is significantly different from zero [42].

### Gini coefficient

The Gini coefficient measures the extent to which individual income is unequally distributed among a population [43]. A Gini index of 0 represents perfect equality (i.e. everyone enjoys the same income level), while an index of 1 or 100% implies inequality [44]. Given the discrete nature of our data, the Gini coefficient is calculated using the following equation [45]:

$$G=(n+1)/n-[2\sum_{i=1}^{n}(n+1-i)x_{i}]/(n\sum_{i=1}^{n}x_{i})$$ (3)

where *G* is the Gini coefficient, *n* the number of observations, and *x_i_* the income size of the *i*^th^ observation, sorted from smallest to largest.

### Methods for estimating change in forest cover and NDVI

To obtain time-series data on MODIS VCF tree-cover for the study area, we first generated a local MODIS 250m tree cover map for each of the 15 years from 2000 to 2014. These data are available from the University of Maryland Global Land Cover Facility (http://glcf.umd.edu/data/vcf/). Annual MODIS VCF data are put together based on 23 16-day composites of the year, with cloud effects being minimized (i.e. pixels with more than 15% cloud cover were excluded from the analysis). Given a total forest area of 9,727 ha and each MODIS pixel having a size of 250m x 250m 1,138 pixels were obtained from the MODIS VCF images over a 15-year time period from 2000 to 2014. Data on the MODIS Normalized Differential Vegetation Index (NDVI) were collected and analysed in the same manner. In this latter case, 6 out of the 23 16-day composites with less than 15% cloud cover were selected with the same spatial resolution of 250x250m. The number of NDVI pixels was 1,148.

### Ethics statement

Part of the analysis presented in this paper is based on interview results with local residents in the study area. Unfortunately, both the University of Amsterdam and the Vrije Universiteit in Amsterdam where this PhD research was undertaken did not have an ethics committee that reviews and approves social science survey research before interviewing took place. The following steps were undertaken to ensure that the interviews were carried out in an ethical manner in accordance with national and international law:

- Before conducting the survey, we had been in contact with the local authority to inform them on the research and were granted legal permission to interview the respondents.
- Respondents in the survey participated on a completely voluntary basis and were not forced, pressured or tricked in any way to answer the questions in the questionnaire.
- Interviews were carried out one-on-one (one interviewer and one respondent) in the safe surrounding of the respondent’s home, and every respondent first had to give his or her explicit verbal consent to the interviewer before being interviewed.
- Before agreeing to participate in the survey, each respondent was informed about (i) the objective of the survey, (ii) the fact that the survey was part of independent scientific research carried out by the Vrije Universiteit Amsterdam with no other commercial or political objectives, (iii) the nature of the questions, i.e. these merely aimed to investigate the socio-demographic and socio-economic status of a representative sample of households in the study area as in an ordinary census and there are no right or wrong answers, (iv) the procedure to guarantee anonymity and confidentiality, i.e. all data and information obtained from the survey would be anonymous and aggregated so that no data and information could ever be traced back to individual respondents, and (v) there would be no follow-up after the interview, and the respondent would not be visited afterwards to ask him or her about his or her answers to the questions.

## Results

### Features of the households participating in the PFES program and the control group

Our sample forms a fair reflection of the ethnic composition of farmers at district level, with 61% of the respondents (n = 185) belonging to the Kinh majority and the rest belonging to ethnic minorities such as K’Ho and Chu Ru. The share of PES participants belonging to the Kinh majority is slightly lower (56%) and significantly higher for non-participants (68%). The Mann-Whitney test is used throughout the paper to examine the significance of observed differences. On average, respondents in our sample are 41 years old, with no significant difference between farmers participating and not participating in the PES scheme. The average household size is 4.7 persons and does not differ significantly between participants and non-participants either. Of these, the number of working family members is 2.7, while the number of financially dependent family members (mostly children and elders) is 2.0 per household. Also here no significant differences exist between the samples of participants and non-participants. Most respondents finished grade 7 (of 12), i.e. the secondary level of Vietnam’s schooling system, but education levels differ significantly between PES participants and non-participants (grade 6.5 and 7.8, respectively).

The average area of agricultural land held by PES participants (1.35 ha per household) is greater than that belonging to non-participants (1.01 ha per household), but not significantly so. Land used for agricultural production, i.e. mainly crop cultivation, comprises both officially and unofficially self-reclaimed land. With regard to crop type, 82% of the households participating in the PES scheme grow coffee as their main crop, while this figure is significantly lower (approximately 20%) for households not participating in PES. Finally, PES participants tend to live on average significantly closer to the forest (21.2 km) than non-participants (26.3 km).

### Costs and benefits of PFES participation

In the study area, participating farmers signed a PFES contract with the local forest owner, the Dran forest management board. The ES buyers are two state-owned entities: the Da Nhim and Xong Pha hydropower plants [40]. In all participating households, one person provides labour to the PES program. When asked about the main reasons for participating in the PES scheme, the most common answer is to earn more income and fulfil their responsibility in protecting the forest. Although the farmers are not the official forest owners, they consider the forest a communal heritage requiring protection and care. Forest protection consists mainly of patrolling the forest for illegal logging and fire prevention and is generally carried out by a group of seven or eight people. Participating farmers visit their assigned forest plot about 6 days per month. The average area of forested land assigned to a household is 28.2 ha, varying between 24.2 and 29.9 ha.

All monetary data in this paper have been converted to 2014 US dollars. The Vietnam Dong (VND) was inflated to 2014 where appropriate using Vietnam’s Consumer Price Index (CPI) and translated into 2014 US Dollars using the World Bank’s exchange rate. For each ha of protected forest participating households receive a fixed payment of US$ 21.3 per year. This payment is calculated by the FDPF and fixed across the entire Da Nhim river watershed [40]. This yields an average annual PES income of US$ 600 per household, ranging from US$ 514 to 636 depending on the allocated area of protected forest. Although this payment rate is considerably higher than payments received in the past, 56% of the respondents consider it to be insufficient, given the costs of PES implementation. These include equipment, medicine, gasoline, and food costs and amount on average to US$ 226 per household per year. According to respondents, expenditures for the last two items, gasoline and food, are highest (33 and 58% of the total costs, respectively). The average net revenue generated from PES in 2014 is therefore US$ 374, which is approximately five times lower than the average net revenue from agriculture, which is US$ 1,905 per household per year. The amount of labour devoted to patrolling the forest, i.e. six days per month, is at the expense of alternative activities such as farming or hired labour, which have an estimated average opportunity cost of US$ 225 per person per year.

### The environmental impacts of PFES

To evaluate the environmental impacts of PES in the study area we used the MODIS VCF images developed by [46]. Overall, the terra MODIS VCF provides three surface components presented as percentages of ground cover: tree, non-tree, and bare cover. In this study, we employed tree-cover as a primary indicator for forest changes. Tree-cover is measured as the percentage of pixels covered by tree canopy. Using spatially explicit time-series satellite images of tree-cover can help identify forested areas [47] and monitor historical forest disturbance and re-growth [48].

**Fig 1:**
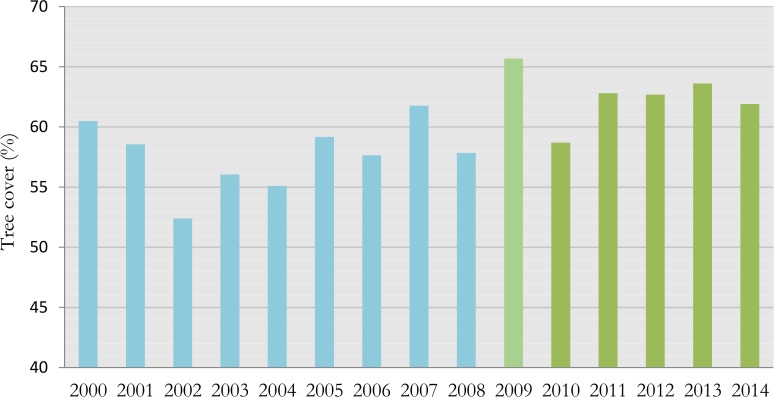
Comparison of mean VCF tree-cover in the study area between the pre-PES period (blue) and during the PES period (green).

Based on the history of PES in Lam Dong, we define a pre-PES period covering the years 2000 to 2008 and the PES period from 2009 to 2014. Data on VCF tree-cover during the pre-PES and PES periods are used for our statistical test. We find a significant 4.9% increase in the average percentage of tree-cover after the introduction of PES in the district Don Duong: from 57.7% to 62.6% (Mann-Whitney z = 19.68; *p*< 0.001). As can be seen from Fig 1, the VCF tree-cover peak (65.7%) occurs in 2009, the first year of the PES pilot phase, while the lowest value is observed just one year earlier in 2008 (57.8%). Tree-cover drops to 58.7% in 2010 to the same level as in 2008, but increases again to 62.8% in 2011 when PES was officially implemented as a national policy in Vietnam, and remains at that level until and including 2014. An important observation from Fig 1 is that tree cover was much more variable before the introduction of PES under previous forestry programs and seems to have stabilized more since 2010.

Besides tree-cover, we also use the MODIS NDVI as a measure of photosynthetic activity and supplementary proxy for vegetation cover. We collected NDVI data for 2007 and 2008 to represent the pre-PES period and for 2013 and 2014 for the PES period. In line with the results for tree-cover, average NDVI in 2013 and 2014 proves to be significantly higher than in 2007 and 2008 (0.77 and 0.75, respectively; Mann-Whitney z = 6.78; *p*-value < 0.001).

In addition to using satellite images, we also organized discussions with local forest authorities and the FPDF about the environmental effects of PES on the local forest ecosystem. Their responses confirm the results of the statistical analysis. According to the Dran forest management board’s report [49], the number of illegal logging incidents in the district Don Duong has dropped by 60% since the official implementation of the PES scheme. Moreover, there has been an improvement in environmental awareness, not only among PES participants, but throughout the community. When asked about their attitude towards forest protection and the significance of forests to society, all respondents stressed the need to protect forests as a vital ecosystem that supplies fresh water and good air quality and prevents flooding. 50% of the households not participating in the PES scheme expressed a willingness to take part in PES if given the opportunity.

### The impacts of PFES on income and local livelihoods

#### Change in income levels

**Fig 2:**
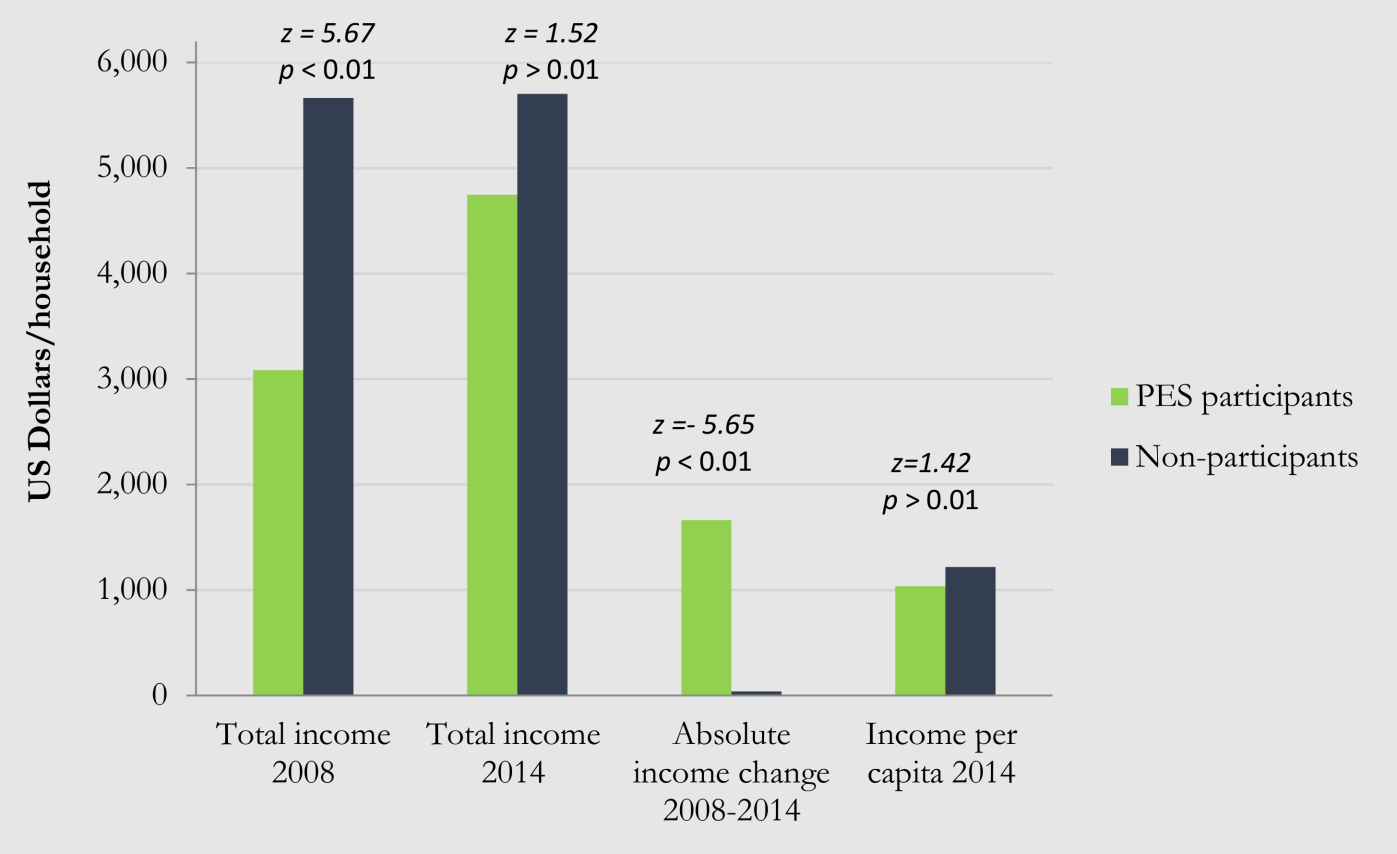
Comparison of changes in gross income of PES participants and non-participants.

The difference in (gross) income between participating and non-participating households was measured at two points in time: in 2008 before PES was piloted in Lam Dong and in 2014, 6 years after PES was introduced in Vietnam in 2009. In order to ensure comparability across the whole time period, only households who had participated in the PES scheme since the pilot phase in 2009 were included in the income analysis. This yields a total of 219 observations (125 PES participants and 94 non-participants). As can be seen from Fig 2, in 2008 the total (gross) income of non-participants (US$ 5,664) was almost twice as high as that of PES participants (US$ 3,086). This reflects the fact that Vietnam’s PES policy prioritizes the involvement of poor and often ethnic-minority farmers, but also demonstrates at the same time the practical difficulty to find a control group of non-participants who has a similar income level as those participating in PES (see for example [37] who reported an income difference of a factor 1.4). However, as the second pair of bars in Fig 2 shows, the income gap between the two groups has narrowed considerably since 2008. In fact, we observe no significant difference in the 2014 income levels between PES participants and non-participants even though the average 2014 income of non-participating households is still higher than that of participating households. Also per capita income of non-participating households is 1.2 times higher than that of participating households in 2014, but again this difference is not statistically significant. Most importantly, PES participants have consistently enjoyed a significantly greater increase in income than non-participants in both absolute (US$ 1,623) and relative terms (38.5%) over the period 2008–2014. Prior to the PES introduction, no statistically significant difference in pre-PES income was found between the majority and minority ethnic groups participating in PES, implying that both groups were equally poor (or rich) before participating in the PES scheme. Six years after their participation in PES, a significant difference was detected between household income of the minority and majority groups, where the latter has a lower income than the former.

Zooming in on agricultural production, i.e. the main source of livelihood for a majority of the inhabitants in the study area, gross agricultural revenues of non-participating households are 1.3 times higher in 2014 than that of their participating counterparts. However, this difference proves to be statistically insignificant. Agricultural production costs, on the other hand, including the purchase of seeds, fertilizer, chemicals and the hiring of labor, are significantly higher for non-participants (1.8 times), resulting in PES participants enjoying slightly higher net agricultural revenues (US$ 1,905 per household) than non-participants (US$ 1,789 per household), but the difference is not statistically significant.

#### Change in livelihoods

Changes in the relative income share of different livelihoods (agriculture, forestry and hired labour) are found to differ significantly between participants and non-participants (see S1 Fig). The average share of agricultural income is consistently higher for non-participants, both before and during PES. Between 2008 and 2014, the share of agricultural income of PES participants decreased by only 2%, and this change is not statistically significant. In contrast, non-participating households show a significant 6% increase in their share of agricultural income. Before the introduction of PES, the share of forestry income was similar for participating and non-participating households (4.0% and 3.4%, respectively). After PES implementation, this share is 21% for participating households and virtually zero for non-participating households. For both groups, the share of hired labor income has declined significantly, but much more so for PES participants (15%) than for non-participants (3%). Finally, other sources of income (e.g. family-run businesses, salaries, and pensions) are observed to be consistently and significantly higher for non-participants than for participants.

#### Change in income inequality

The magnitude of income inequality between PES participants and non-participants was assessed by calculating the Gini coefficients for the two groups. The current (2014) income distribution is more equal among PES participants than non-participants. The same finding is reported in a study by [43] related to one of the largest PES schemes in the world in China. Before the implementation of PES, the Gini coefficient was slightly higher for participants (0.472) than non-participants (0.442), implying a more skewed (unequal) income distribution in the sample of participants. This changed considerably after PES implementation and the Gini coefficient for households taking part in the PES scheme is 0.380, 6 years after the introduction of PES. This implies a 20% reduction in income inequality. The result for the non-participants shows a much lower decrease in income inequality of 3%, from 0.442 in 2008 to 0.427 in 2014. Calculating the Gini coefficient for the whole sample population, consisting of both PES participants and non-participants, a 17% reduction in income inequality (from 0.489 to 0.404) is found.

#### Determinants of changes in household income levels

A treatment effects model was estimated to account for possible endogeneity between the decision to participate in the PES scheme and household income and simultaneously investigate the factors that determine (i) farmers’ likelihood to participate in PES and (ii) household income change. The robustness of the treatment effects model was tested by comparing the results for the *relative* income change with those obtained from the same model estimated for the *absolute* change in income in Table 1. The effects appear to be exactly the same and hence robust. The estimated models are furthermore highly significant as can be seen from the outcome of the Wald test. The outcome of λ (the product of the correlation between the two error terms ρ and the standard error of the OLS regression σ) confirms the existence of a significant correlation between (i) and (ii) at the 1% level and hence the relevance of using the treatment effects model.

**Table 1:** Results of the treatment effects model.

	Relative income change		Absolute income change	
Explanatory variable	Coefficient estimate	Std. error	Coefficient estimate	Std. error
***Outcome model*** *(dependent variable*: *change in household income)*				
*Intercept*	-0.533***	0.205	-97.035***	33.874
Ethnicity (= 1 if Kinh)	0.014	0.066	1.896	11.021
Years living in area (= 1 if > 20 years)	-0.175	0.116	-17.673	19.614
Years in PES scheme	0.039*	0.024	6.433*	3.834
Number of working family members	0.034*	0.021	6.993**	3.279
Number of dependent family members	0.027	0.024	2.179	3.963
Agricultural land size (ha)	0.041**	0.021	9.124***	3.339
Main crop type (= 1 if coffee)	-0.001	0.062	0.222	9.632
PES participation (= 1 if yes)	0.814***	0.204	116.269***	33.874
***Selection model*** *(dependent variable*: *PES participation)*				
*Intercept*	1.431***	0.456	1.431***	0.456
Ethnicity (= 1 if Kinh)	0.056	0.230	0.056	0.230
Years living in area (= 1 if > 20 years)	0.333	0.336	0.333	0.336
Age (years)	-0.013*	0.008	-0.013*	0.008
Distance to forest (km)	-0.016**	0.007	-0.016**	0.007
Number of dependent family members	0.021	0.066	0.021	0.066
Pre-PES income level (US$/household)	-0.004***	0.001	-0.004***	0.001
Pre-PES forestry activities (= 1 if yes)	-0.349*	0.196	-0.349*	0.196
***Model statistical summary***				
Wald χ^2^	37.73		33.94	
Prob>χ^2^	0.0001		0.0004	
Λ (Lamda)	-0.392***		-75.054***	
Rho (Correlation coefficient)	-0.840		-0.948	
Model standard deviation	0.466		79.151	
N	264		264	

*, **, *** statistically significant at 10%, 5% and 1% respectively.

The selection model shows that respondent age, distance to the forest, pre-PES income and pre-PES forestry activities have a statistically significant effect on a farmer’s decision to participate in PES. A negative relationship is observed for all these explanatory variables. Older respondents are less likely to participate in the PES scheme than younger respondents. In line with prior expectations and comparable to the findings reported in [50], increasing distance significantly decreases the likelihood of PES participation. Similarly, households involved in forestry programs before are found to be less likely to participate in PES, possibly because of the low payment rates received in the past. Families who earned less money prior to PES implementation, and hence had lower opportunity costs, tend to be more motivated to participate in the scheme as a means of acquiring additional income.

With regards to the change in household income, a significant positive effect is detected for the following variables: PES participation, the number of years a farmer is in the PES scheme (since not all respondents have been participating since the beginning), the number of working family members, and agricultural land size. Most findings are in line with prior expectations. Household participation in PES has a significant positive influence on the change in household income whilst controlling for other explanatory factors. A positive relationship is also detected for the number of years a farmer participates in the PES scheme: the longer the farmer participates, the more absolute and relative income changes. Similarly, an increase in agricultural land size or the amount of family labour helps to raise household income. No significant relationships are found in the two regression models for the number of dependent family members, the number of years that farmers have been living in the study area, whether or not households belong to the Kinh majority or a minority group, and main crop type. Control was also included for the 4 villages where the survey was carried out, but none of the dummy variables were statistically significant, most probably due to the fact that the selected villages are very much alike, and are therefore not included in the model presented in Table 1.

## Discussion

This is the first study to examine the impacts of Vietnam’s PES scheme on both local forest quality and the livelihoods of participating households. Changes in forest tree-cover and household income levels and their distribution are measured before and after PES implementation. Earth observations to assess forest ecosystem impacts were used over a period of 15 years, while changes in income were measured over a period of seven years across PES participants and non-participants as a control group. The study reveals that between the pre-PES period and the period since PES was introduced in the study area, a statistically significant increase in average percentage tree-cover was observed while the absolute and relative changes in income proved to be significantly higher for participating households than non-participating households. The average income level of participating households has increased by 45% since the introduction of PES in Vietnam. After PES, also a more pronounced improvement in income distribution can be observed among those participating in PES compared to those not participating in the PES scheme.

Equally important, this study shows furthermore that whilst controlling for other relevant socio-economic trends and characteristics, changes in income levels are driven significantly by the number of years families participated in the PES scheme. The positive impact on income increases the longer households participate. As such, we were able to isolate the impact of PES participation on income change and conclude that this has been a significant factor behind income growth. The contribution of forestry income in particular has increased significantly since 2009 as a result of PES participation, nowadays ranking as the second most important income source after agriculture. Although generating the largest share of household income, income flows from agriculture are less stable. Agricultural income depends on various exogenous factors, including weather conditions, changes in the price of inputs such as fertilizer and pesticides, and changes in market selling prices. Farmers in the study area reported to be generally poorly informed about market conditions and to have limited negotiating power with traders. In contrast, the periodic PES payment is stable and helps families to pay their children’s tuition fees, daily living expenses and outstanding loans. However, an important concern relates to the longer term financial viability of the PES scheme in view of the fact that the contracts between forest owners and farmers are renewed annually, with payments largely depending on the involvement of and overall coordination by the FPDF. Combined with the legal constraints on protected forested land conversion, this introduces some degree of uncertainty about future income security.

Finally, a few limitations of this study should be mentioned. First, because our research focuses on the district of Lam Dong, some caution should be exercised in generalizing the results. The sample is considered representative for the district and province, but not necessarily for the country as a whole. Further research in other areas where PES has been rolled out in Vietnam is needed. Second, it should be borne in mind that the data used for the livelihood analysis are based on self-reported recall information obtained from farmers during the field survey. This may have introduced a degree of unreliability and possibly strategic reporting bias. The recall information is hard to verify since there are no official statistical records of household incomes in the study area. To overcome this limitation, we asked farmers to report their income generated from every single income source and we calculated the total household income based on this detailed breakdown. We also compared our estimates with results from studies conducted previously in the same district of Don Duong and found comparable values. Third, due to measuring difficulties and time constraints, we were unable to assess other potential factors of influence on the observed impacts. These include, for example, the role of social coherence on PES participation. Last but not least, it is worth mentioning that the results related to the economic impacts are more statistically convincing than those of the environmental impacts, due to several reasons. First, given the 5 years of PES implementation in the study area, it seems still early to analyze and draw a convincing conclusion on the environmental impacts of PES, as the scheme needs long-term monitoring to validate their contribution to the provision and maintenance of ecosystem services [32]. As commented by [5] it is very costly and technically difficult to identify a clear link between forest protection and the provision of ES. Second, it was not possible to compare forest cover inside and outside the PES area in the same watershed in view of the fact that most forests in the Da Nhim watershed are already in the PES scheme. Third, it could be argued that the 250m spatial resolution of the MODIS images employed in this study for the environmental impact assessment is perhaps somewhat coarse for an accurate reflection of the scale at which the human-induced land-use changes occur in this specific local case study.

## Supporting information

S1 Table General household characteristics across the 4 selected villages. (PDF)

S1 Text Survey form for PES participants. (PDF)

S2 Text Survey form for non-participants. (PDF)

S1 File Anonymized data set of the survey. (XLSX)

S1 Fig Relative share of different income sources in total household income for PES participants and non-participants, before and after PES implementation. (PDF)
