# Supplementary material for: Do payments for forest ecosystem services generate double dividends? An integrated impact assessment of Vietnam’s PES program
Source: PLoS One. 2018 Aug 1;13(8):e0200881. doi: 10.1371/journal.pone.0200881 (PMC6070196; doi:10.1371/journal.pone.0200881)
Supplement: S1 Table — (PDF) [file pone.0200881.s001.pdf]

S1 Table. General household characteristics across the 4 selected villages.

Variable	Village			
	**Hamashing**	**Lam Tuyen**	**PhuThuan**	**Ma Rang**
Share belonging to Kinh majority	0.63	0.63	0.59	0.61
Mean education level (years)	6.98	7.08	6.84	7.02
Mean age (years)	40.72	40.75	39.46	40.88
Share living >20 years in study area	0.92	0.92	0.95	0.92
Mean distance to the forest (km)	22.40	22.28	22.21	21.97
Mean household size (number of persons)	4.67	4.67	4.69	4.73
Mean number of working household members	2.59	2.61	2.62	2.67
Mean number of dependent household members	2.08	2.06	2.07	2.05
Share coffee as main crop	0.78	0.77	0.79	0.74
Mean agricultural land size (ha)	1.18	1.15	1.20	1.13
Mean total income before PES (US $/household)	3,778	3,761	3,401	3,912
