# Supplementary material for: Do payments for forest ecosystem services generate double dividends? An integrated impact assessment of Vietnam’s PES program
Source: PLoS One. 2018 Aug 1;13(8):e0200881. doi: 10.1371/journal.pone.0200881 (PMC6070196; doi:10.1371/journal.pone.0200881)
Supplement: S1 Text — (PDF) [file pone.0200881.s002.pdf]

S1Text. Survey at household level for PES participants

INTRODUCTION

This interview is part of independent scientific research for my PhD. The purpose of this interview is to discuss about the impacts of PFES in Lam Dong on household income and livelihoods. Insights from this interview will be used solely for scientific purposes. The expected outcome is a scientific report. All information obtained from this interview will be treated completely confidential and anonymous. No names will be used in the final report.

Name of interviewee: ……

Ethnicity: ....

Age: …………………………………

Position in the family: …………………………………………………………….........................

Educational level: ……………………………………………………………………………

Address: ……………………..

Phone: …………………………………………

Interviewer: ………………………………………………………………………………….........

Date of interview: ………………………

Time: …………………..

Part 1: General household characteristics

1. *How long have you been living here?* ....years
2. *Household members and labours:*

**Description**		**Persons**
Q2-1	Number of household members	
Q2-2	Number of members who are working	
Q2-3	Number of members having monthly salary	
Q2-4	Number of children below 15 years old	

Q2-5	Number of members older than 65 years

3. *Agricultural and forested land:*

	Before PES	PES phase (2014)
Agricultural land (hectare)		
Forested land (hectare)		

4. *How long have you been working in the forestry sector?*

   ....*years*

Part 2: Payment for Forest Environmental Services

5. *When did you start participating in the PES programme?*

Year: …………………………………………

6. *What are your 3 main reasons to participate in PES?*

a. More income for your family
b. General responsibility in forest protection
c. Increased forest quality and value
d. More jobs
e. Improved soil quality
f. Technical assistance
g. Land tenure
h. Social relations
i. Others, namely: …………………………………………………………………..

7. *Did you participate in one of the below forestry programs before PES (and when)?*

 The 661 Program (year: ....)
 The Provincial Budget Program (year: ....)
 The 304 Program (year: ....)
 The Forest for Livelihood Improvement in the Central Highlands Program (year: …..)
 Others, namely: ……………………………………… (year: …………………..................)
 Not participating in any programs

8. About the non-PES payment programs:

Q8-1 How many hectares of your forest were registered under that program? ....hectare

Q8-2 What is the payment rate of that program?

................thousands VND/hectare OR ....thousands VND/year

Q8-3 What is the current status of that program?

 Finished (since year: ……………) Still on going

9. PES contract:

Q9-1 Did you sign a contract in order to participate in PES?

 Yes No

Q9-2 What is the type of contract?

 Grouped household contract Individual household contract

Q9-3 Who did you sign the contract with?

....

Q9-4 What is the contract duration?

.... years

10. According to the contract, what are your main responsibilities as the ES providers?

....

11. In case of non-compliance, what are the forms of penalty?

 Contract termination

 No payment received

 Others, namely:

....

12. Payment rates of all forestry programs (before and after PES):

	Before PES	2014
Name of the program		
Payment frequency (times/year)		
Payment rate for each time (thousands VND/time)		
Payment mode (cash/in-kind/mixed)		

13. *What do you think about the current payment rate/hectare (2014)?*

 Reasonable Low High

14. *What is the desirable payment rate according to you?*

.... thousands VND/hectare

Part 3. Household income and labour structure

15. *What are the main income sources in your family in the following phases?*

	**Before PES**	**PES phase (2014)**
Agriculture (Cropping/Husbandry)		
Forestry		
Aquaculture		
Salary, pension, subsidies		
Others, namely: ………………………………………..		

16. *What is the total income level of your family in the following phases?*

Q16-1 Before PES:

.... VND/month OR .... VND/year

Q16-2 PES phase (2014):

.... VND/month OR .... VND/year

17. *Please specify the income level from agriculture and forestry:*

	Agriculture generate income (VND/year)	Forestry generated income (VND/year)
Before PES		
PES phase (2014)		

18. *What are the other income sources from forestry, besides PES?*

 Legal/illegal timber extraction

 Non-timber forest products

 Others namely...

□ No other income

19. Can you estimate the income generated from those sources in the following phases?

Q19-1 Before PES:

.... VND/month OR .... VND/year

Q19-2 PES phase (2014):

.... VND/month OR .... VND/year

20. What are the 3 main types of expenses in your family?

a. Daily expenses
b. Education
c. Health care
d. Buying household equipment
e. Investment in agriculture
f. Investment in forestry
g. Others, namely: ....

21. What is the labour structure of your family in the following phases?

	Number of labour (persons)		Time spent (days/month)	
	Agriculture	Forestry	Agriculture	Forestry
Before PES				
PES phase (2014)				

22. What are the other benefits, in addition to the payment, when participating in PES?

22.1 Technical training:

□ No □ Yes (........................ times/year)

22.2 Other benefits:

Rice provision: □ No □ Yes (........................kg/year)

Seeds/plants: □ No □ Yes (........................kg/year)

Loans: □ No □ Yes (........................VND/year)

Costs of forest protection/forestation

23. (Labour costs) Do you have to hire external labour?

□ No □ Yes (................ persons/forest visit)

24. *(Time costs) Can you estimate the amount of time spent for taking care of the forest?*

- Q24-1 Number of labour: ....persons/visit
- Q24-2 Labour cost (per day per person): ....VND/day/person
- Q24-3 Number of forest visit: ....times/month
- Q24-4 Number of hour per visit (excluding travel time): ....hours/visit
- Q24-5 Average distance from home to the forest: ……………………………km
- Q24-6 Distance from home to the nearest forest: ……………………………km
- Q24-7 Distance from home to the farthest forest: ……………………………km
- Q24-8 Travel time (between home and the forest, one way): ....hours/person
- Q24-9 Transport means: ....

25. *Can you estimate the costs associated with PES (equipment, gasoline, food, medicine)?*

Item	Cost (VND/year)
Gasoline	
Knife	
Light(s)	
Gloves	
Blanket(s)	
Food	
Medicine	

26. *Other costs, if any:*

....

27. *Can you estimate the cost of agricultural production?*

Item	Cost (VND/year)
Seeds/Plants	
Animals	
Equipment	
Fertilizer	
Pesticides/ Herbicides	

Facility investments	
Others, namely:	
Total	

28. *How do you often spend the money from PES (3 choices)?*
    - To cover household daily expenses
    - To cover educational and healthcare expenses
    - To cover agricultural costs
    - To cover forest protection costs
    - To contribute to a collective fund that will be used for the whole community
    - Others, namely: ……………………………………………………..

Other PFES related issues

29. *How do you receive PES money?*
    - Home delivery
    - At a money collecting point, namely:
    ....
    - Via bank transfer
    - Others, namely: ......…………………………………………………………
30. *In case there is a collecting point, what is the distance between home and that place?*
    ....km
31. *How long does it take from home to there?* ....hours OR days (one way)
32. *Within your group, who is in charge of writing the monitoring reports?*
    - Everyone takes turn
    - One assigned person does it all the time (who: ……………………………….)
33. *How many times do you have to submit a monitoring report to the authority?*
    ....times per month OR per year
34. *How much time does it cost you to finish a report?*
    ....hour(s) per report

Part 4. Environmental impacts and environmental awareness

35. Q35-1 Do you think that forests bring benefits to our life?
     Yes No

Q35-2 If Yes, can you explain why/list down some benefits:

....

36. Q36-1 Do you think that PES helps protecting forest in a better/more effective way?

 Yes No

Q36-2 If Yes, can you explain why/list down some reasons:

....

37. What would you do with your forest if PES does not exist?

....

38. Can you qualitatively evaluate the changes in the following issues since the PES introduction?

Issue	Increase	Unchanged	Decrease
Q38-1 Total forest area			
Q38-2 Number of fires/year			
Q38-3 Number of illegal logging/year			
Q38-4 Soil quality			
Q38-5 Responsibilities of forest users as ES providers			
Q38-6 Regulations about penalty in case of non-compliance			
Q38-7 Money spent on forestry practices			
Q38-8 Accessibility to governmental loan			

39. Do you have any recommendations on how to improve PES?

 No

 Yes, e.g...

This is the end of the interview. Thank you for your participation!
