# Supplementary material for: Do payments for forest ecosystem services generate double dividends? An integrated impact assessment of Vietnam’s PES program
Source: PLoS One. 2018 Aug 1;13(8):e0200881. doi: 10.1371/journal.pone.0200881 (PMC6070196; doi:10.1371/journal.pone.0200881)
Supplement: S2 Text — (PDF) [file pone.0200881.s003.pdf]

S2 Text. Survey at household level for non-participants

INTRODUCTION

This interview is part of independent scientific research for my PhD. The purpose of this interview is to discuss about the impacts of PFES in Lam Dong on household income and livelihoods. Insights from this interview will be used solely for scientific purposes. The expected outcome is a scientific report. All information obtained from this interview will be treated completely confidential and anonymous. No names will be used in the final report.

Name of interviewee: …………………Ethnicity: ....

Age: …………………………………

Position in the family: ………………………………………………………………...............................

Educational level: ………………………………………………………………………………......

Address: ………………………...

Phone: …………………………………………

Interviewer: ……………………………………………………………………………………..................

Date of interview: ………………………Time: …………………...

Part 1: General household characteristics

1. How long have you been living here? ....years
2. Household members and labours:

**Description**	**Persons**
Number of household members	
Number of members who are working	
Number of who are not working	

3. Agricultural land and main crops:

	**Year 2008**	**Now (2014)**
Main crops		
Agricultural land (hectare)		

Part 2: Past experience of working in forestry sector

4. Had your family ever participated in the following forestry programs?
   a. PES (in year: ………………………………………………………………
   b. The 661 Programme (in year: ………………………………………………………………………………
   c. The Provincial Budget Programme (in year: ………………………………………………………………)
   d. The 304 Programme (in year: ………………………………………………………………………………)
   e. Others, namely: ……………………………………… (in year: ……………………………)
   f. None
5. About the program specified in Q.4:

   Forest area assigned: ………………………………… (ha)

   Payment rate: ………………………………………… VND/ha/year
6. When did your participation end? ……………………………………………………… (year)
7. What are the main reasons of not participating anymore in the program specified in Q.4?
   a. Lack of labour
   b. Lack of health
   c. Not selected
   d. Low payment
   e. Others, namely: ……………………………………………………………………..

Part 3. Household income and labour structure

8. *What are the main income sources in your family in the following phases?*

	**Year 2008**	**PES phase (2014)**
Agriculture (Cropping/Husbandry)	☐	☐
Forestry	☐	☐
Aquaculture	☐	☐
Salary, pension, subsidies	☐	☐
Others, namely: …………………………………………..	☐	☐

9. *What is the income level of your family in the following phases?*

Q16-1 Before PES:

.... VND/month OR .... VND/year

Q16-2 PES phase (2014):

.... VND/month OR .... VND/year

10. Please specify the income level from agriculture and forestry:

	Agriculture generate income (VND/year)	Forestry generated income (VND/year)
Year 2008		
PES phase (2014)		

11. What are the main types of expenses in your family?

a. Daily expenses
b. Education
c. Health care
d. Buying household equipment
e. Investment in agriculture
f. Investment in forestry
g. Others, namely: ....

12. What is the labour structure of your family in the following phases?

	**Number of labour (persons)**		**Time spent (days/month)**	
	Agriculture	Forestry	Agriculture	Forestry
Year 2008				
Now (2014)				

13. *Can you estimate the cost of agricultural production?*

**Item**	**Cost (VND/year)**
Seeds/Plants	
Animal	
Equipment (grass machine, pump, spray, pines)	
Fertilizer	
Pesticides/ Herbicides	
Facility investments	
Others, namely:	

Part 4. Environmental impacts and environmental awareness

14. *Do you think that forests bring benefits to our life?*

Yes No

If Yes, can you explain why and list some benefits:

....

15. *Can you qualitatively evaluate the changes in the following aspects since 2009?*

**Issue**		**Increase**	**Unchanged**	**Decrease**
Q15-1	Total forest area			
Q15-2	Number of fires/year			
Q15-3	Number of illegal logging/year			
Q15-4	Soil quality			

16. *If your family is offered to participate in PES, are you willing to join and why?*

 Yes (because: ....

 No (because: ....

This is the end of the interview. Thank you very much for your time and participation!
