# Supplementary material for: Do payments for forest ecosystem services generate double dividends? An integrated impact assessment of Vietnam’s PES program
Source: PLoS One. 2018 Aug 1;13(8):e0200881. doi: 10.1371/journal.pone.0200881 (PMC6070196; doi:10.1371/journal.pone.0200881)
Supplement: S1 Fig — (PDF) [file pone.0200881.s005.pdf]

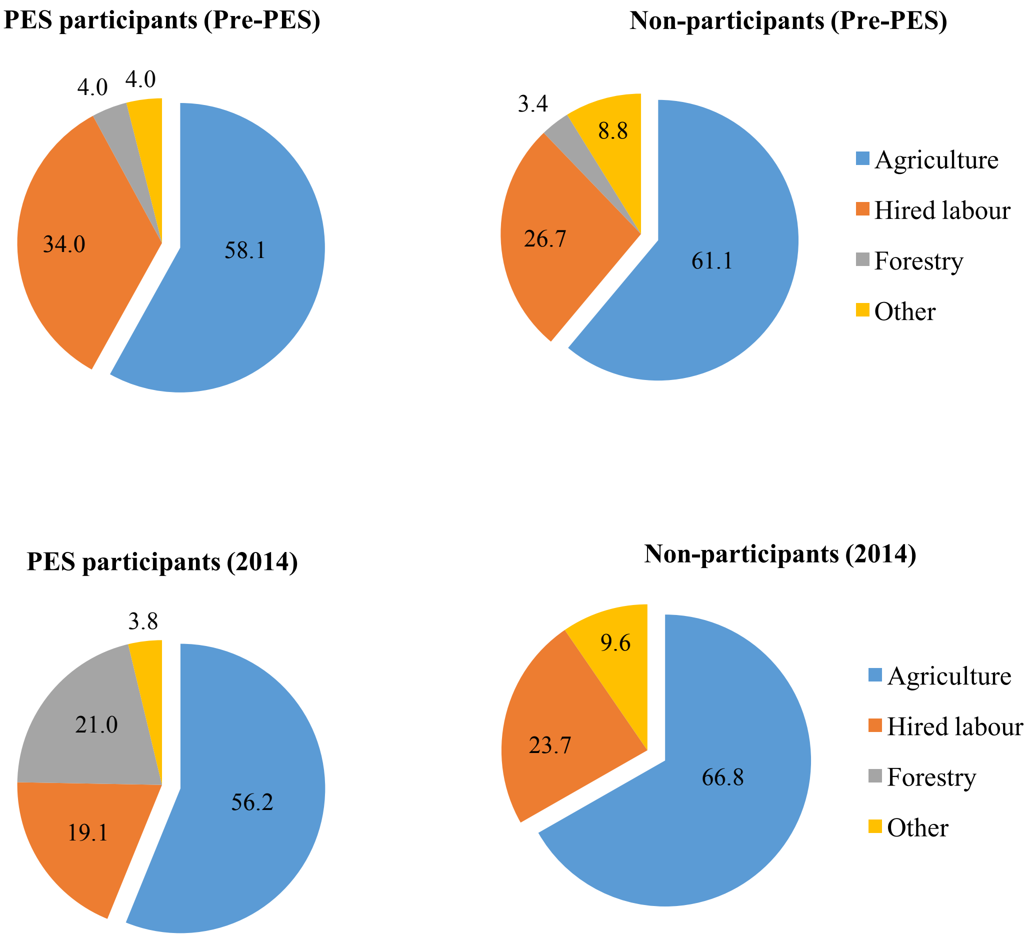

S1 Fig. Relative share of different income sources in total household income for PES participants and non-participants, before and after PES implementation.
